# NF-κB signaling pathway mechanism in cow intertoe skin inflammation caused by *Fusobacterium necrophorum*

**DOI:** 10.3389/fcimb.2023.1156449

**Published:** 2023-04-21

**Authors:** Hefei Zhang, Jiasan Zheng, Yue Sun, Chunxue Yang, Yang Yue

**Affiliations:** College of Animal Science and Veterinary Medicine, Heilongjiang Bayi Agricultural University, Daqing, China

**Keywords:** skin explants, bacterial infection, *Fusobacterium necrophorum*, NF-κB, inflammatory response

## Abstract

**Background:**

*Fusobacterium necrophorum* is the main pathogen inducing bovine foot rot. The infected site is often accompanied by a strong inflammatory response, but the specific inflammatory regulatory mechanism remains unclear.

**Aim:**

A cow skin explants model was established to elucidate the mechanism of *F. necrophorum* bacillus causing foot rot in cows, and to provide reference for future clinical practice.

**Methods:**

Cow intertoe skin explants were cultured *in vitro*, and *F. necrophorum* bacteria solution and nuclear factor-κB (NF-κB) inhibitor BAY 1-7082 were added to establish an *in vitro* infection model. Hematoxylin and eosin staining, terminal - deoxynucleotidyl transferase mediated nick end labeling, and immunohistochemistry were used to detect the pathological changes of the skin explants infected with *F. necrophorum*, the degree of tissue cell apoptosis, and the expression of the apoptosis-related protein Caspase-3, respectively. RT-qPCR, Western blot, and ELISA were used to detect the activation of the NF-κB pathway and inflammatory cytokines by *F. necrophorum*.

**Results:**

The intertoe skin structure of cows infected with *F. necrophorum* changed with different degrees of inflammation, and the degree of tissue cell apoptosis was significantly increased (*P* < 0.01). In addition, infection with *F. necrophorum* significantly increased the phosphorylation level of IκBα protein and up-regulated the expression level of NF-κB p65. The high expression and transcriptional activity of NF-κB p65 significantly increased the expression and concentration of the inflammatory cytokines TNF-α, IL-1β, and IL-8, thus inducing the occurrence of an inflammatory response. However, inhibition of NF-κB p65 activity significantly decreased the expression of inflammatory factors in the intertoe skin of cows infected with *F. necrophorum*.

**Conclusion:**

*F. necrophorum* activates NF‐κB signaling pathway by increasing the expression of TNF‐α, IL‐1β, IL‐8 and other inflammatory factors, leading to foot rot in dairy cows.

## 1 Introduction

Bovine foot rot has also been referred to as interdigital necro-bacillosis, interdigital pododermatitis, interdigital phlegmon, foul in the foot, and foot abscess (Van Metre, 2017). It is a highly infectious contact disease mainly caused by *Fusobacterium necrophorum*, *Dichelobacter nodosus*, and other pathogenic microorganisms (single or mixed infection) of interdigital skin and deep soft tissue necrosis (Clifton et al., 2019; Kontturi et al., 2019). The average incidence rate of bovine foot rot has been calculated as 17%–25% (Kontturi et al., 2019). In a recent outbreak of bovine foot rot in Finland, the incidence rate in 203 cows was almost 30%. The disease does not only has a high incidence, but is also widespread, with occurrences reported in North America, South America, Australia, New Zealand, Europe, Asia, and Africa (Kontturi et al., 2020). The skin and deep soft tissues between the toes of cows with foot rot become red, swollen, and necrotic, leading to lameness, accompanied with varying degrees of appetite loss and fever. This has a serious impact on the milk production, productivity, animal welfare, and farm economic benefits.

Foot rot often leads to local or even systemic inflammatory reactions. A recent study by Kontturi et al. found that serum amyloid A and haptoglobin were elevated in cows with naturally occurring foot rot, resulting in an acute phase response (Kontturi et al., 2020). Serum amyloid A and haptoglobin can be used as two main acute phase proteins to determine the severity of inflammation in infected foot rot (Kujala et al., 2010; O'Driscoll et al., 2015). Inflammation is a basic response of the body to repair damage and immune system defense. Under normal circumstances, it can help the body remove harmful factors and promote repair, but excessive inflammation will aggravate tissue damage (Allard et al., 2018). Nuclear factor-κB (NF-κB) is a widespread transcriptional regulator that plays a key role in regulating the development and homeostasis of the immune system, and in coordinating inflammatory responses (Mitchell and Carmody, 2018). Studies have shown that NF-κB can induce the overexpression or sustained expression of at least 27 different inflammatory target genes (Baker et al., 2011). In the classical pathway, Iκbα is dissociated from the Iκbα-NF-κB dimer by phosphorylation of active IκB kinase (Hu et al., 2016), followed by NF-κB transfer into the nucleus to activate the transcription of pro-inflammatory genes through the synthesis of cytokines such as TNF-α and IL-1β (Li et al., 2016). This leads to an inflammatory response in the body. In the present authors’ previous proteomic studies, a differential expression of proteins in cows with foot rot was found to be involved in the regulation of TNF-α and NF-κB signaling pathways (Jiasan et al., 2016). Further studies confirmed that the inflammatory response mediated by the NF-κB signaling pathway plays an important role in the pathogenesis of bovine foot rot. However, the detailed mechanism of the activation of this pathway is still unclear. The purpose of this study was to investigate the possible mechanism behind *F. necrophorum* inducing inflammation in the intertoe skin of dairy cows, and to provide new insights into the regulatory mechanism of inflammation in bovine foot rot.

## 2 Materials and methods

The study protocol was approved by the Ethics Committee on the Use and Care of Animals, Heilongjiang Bayi Agricultural University (Daqing, China). The animal studies were performed in accordance with the Guiding Principles of Animals adopted by the Chinese Association for Laboratory Animal Sciences.

### 2.1 Activation and culture of *F. necrophorum*

The BNCC336998 strain of *F. necrophorum* was purchased from BeNa Culture Collection (Xinyang, China) and stored in the Veterinary Surgery Laboratory of Heilongjiang Bayi Agricultural University (Daqing, China). Thawed BNCC336998 was inoculated in Fastidious Anaerobe Broth medium for 48 h, and bacterial growth was observed by Gram staining. The absorbance of *F. necrophorum* at optical density600 was detected by an enzyme-label when the growth of *F. necrophorum* was at the logarithmic stage, and the concentration of the bacterial solution was obtained according to the Wheat - than kit (Solarbio Science & Technology Co., Beijing, China). The concentration of bacterial solution was adjusted to 1 × 108/mL by adding Dulbecco’s Modified Eagle Medium (DMEM)/F-12 1:1 medium (Thermo Fisher Scientific (China) ltd, Shanghai, China).

### 2.2 Model construction of *F. necrophorum* explant infection in the intertoe skin of dairy cows

Healthy cow hooves were collected from local slaughterhouses. After repeated cleaning and disinfection, the interdigital skin hair was removed with a sterile scalpel and scissors, then all of the interdigital skin was removed and immediately immersed in transport medium (100 mL transport medium: Penicillin-Streptomycin-Amphotericin B Solution, 100× [Beyotime Institute of Biotechnology Co., Shanghai, China] 1 mL, L-glutamine solution [Solarbio Science & Technology Co., Beijing, China] 1 mL, gentamycin sulfate [Solarbio Science & Technology Co., Beijing, China] 5 µL, the rest are DMEM/F12 1:1 medium Thermo Fisher Scientific (China) ltd, Shanghai, China]), and transported back to the laboratory in ice.

A sterile scalpel and scissors were used to remove the excess subcutaneous fat from the interdigital skin on a super-clean work table. The interdigital skin explants were collected aseptically with a 8-mm punch biopsy (Tedpella Inc., California, USA) and immersed in a washing medium preheated to 37°C The explants were washed 3 times (15 min each) at 25°C in 100 mL of washing medium (Penicillin-Streptomycin-Amphotericin B Solution, 100× [Beyotime Institute of Biotechnology Co., Shanghai, China] 1 mL, gentamycin sulfate [Solarbio Science & Technology Co., Beijing, China] 4 µL, the rest are DMEM/F-12 1:1 medium).

The washed skin explants were fixed in the inner chamber of the cell culture chamber, and 2 mL of tissue medium (in 100 mL of washing medium, Penicillin-Streptomycin-Amphotericin B Solution, 100× [Beyotime Institute of Biotechnology Co., Shanghai, China] 1 mL, L-glutamine solution [Solarbio Science & Technology Co., Beijing, China] 1 mL, gentamycin sulfate [Solarbio Science & Technology Co., Beijing, China] 10 µL, fetal bovine serum [Thermo Fisher Scientific (China) ltd, Shanghai, China] 10 mL, DMEM high glucose medium [Thermo Fisher Scientific (China) ltd, Shanghai, China] 65.99 mL, allowance: DMEM/F-12 1:1 medium) was added to the outer chamber, and 1 mL of 1 × 108/mL of *F. necrophorum* bacteria solution was added to the inner chamber. Tissue medium was then added to the epidermal and dermal junction of the skin explants to form an alternate culture system of gas and liquid phases. The explants were cultured in a humidified incubator at 37°C and 5% CO2, and the liquid was changed once every 12 h. Two skin explants were collected at 0, 12, 24, 36, 48, and 72 h after infection. One was frozen in a refrigerator at −80°C to determine *F. necrophorum* infection in the skin explants. The other was fixed in 4% paraformaldehyde fixation solution (Servicebio Biological Technology Co., Ltd. Wuhan, China) for the evaluation of model activity.

To evaluate model activity, hematoxylin and eosin (HE) staining under different fields was first observed to evaluate the pathological changes of the skin explants. Five high-magnification fields were then randomly selected by TUNEL staining and an immunohistochemical method to detect the activity of cells in the epidermis and dermis, and the positive expression of the apoptosis-related protein Caspase-3.

### 2.3 Bacterial genomic DNA extraction and polymerase chain reaction (PCR) identification of skin explants infected with *F. necrophorum*

In order to verify the *F. necrophorum* infection of skin explants, DNA from the skin explants was extracted according to the instructions of the TIANamp Bacteria DNA Kit (TIANGEN Biotechnology Co., Beijing, China), and a PCR reaction was performed under the following conditions: 95°C 5 min; 94°C, 1 min; 54°C, 1 min; and 72°C, 1.5 min; for 30 cycles. The reaction finished at 72°C for 10 min and was then cooled to 4°C. The amplified products were identified by 1% agarose gel electrophoresis. The primer sequence is shown in **Table 1**.

**Table 1:** Primer sequence of the gene.

Gene	primer sequence (5’-3’)	Length(bp)
*Fusobacterium necrophorum* 16S-rRNA	F: GCTCTCCTGCTTGTTTATTTC	1030
	R: GATCTTTGTTGGAAGCGAGT	

### 2.4 Evaluation of the model activity of *F. necrophorum* explant infection in the intertoe skin of dairy cows

Paraffin sections were routinely prepared (Sy and Ang, 2019), and 4-μm skin tissue sections were stained according to the HE Staining Kit (Solarbio Science & Technology Co., Beijing, China) and TUNEL Apoptosis Assay Kit (Beyotime Institute of Biotechnology Co., Shanghai, China). The pathological changes of the skin explants and the number of apoptotic cells were then observed. The expression of the apoptosis-related protein Caspase-3 was detected by immunohistochemistry, and the dilution ratio of the Caspase-3 primary antibody (Bioss Biotechnology co., ltd, Beijing, China) was 1:300. For the assessment of the apoptosis rate, five epidermal and dermal fields of 400× were randomly selected. The apoptotic cells and total cells under the field were counted using Fiji/ImageJ-win 64 software (https://fiji.sc/). The apoptosis rate was calculated according to the formula apoptosis rate = number of apoptotic cells/total number of cells × 100%.

### 2.5 Concentrations of TNF-α, IL-1β, and IL-8 in the medium detected by enzyme-linked immunosorbent assay

Skin explants were collected and cleaned as described above, then treated with 1 × 108/mL *F. necrophorum* bacteria solution and/or 50 mM BAY 11-7082 (Beyotime Institute of Biotechnology Co., Shanghai, China) for 24 h. The culture medium was collected and centrifuged at 300× g for 20 min. The supernatant was extracted using an enzyme-linked immunosorbent assay kit (TNF-α: MM-190301; IL-1β: MM-3694901; IL-8: MM-3695101, Meimian Industrial Co., Ltd, Jiangsu, China) and was used to determine the concentrations of inflammatory cytokines TNF-α, NF-κB, IL-1β, and IL-8 in the medium.

### 2.6 RNA extraction and real-time quantitative PCR (RT-qPCR)

Total RNA from skin explants after 24 h treatment was extracted using Trizol (Thermo Fisher Scientific (China) ltd, Shanghai, China) according to the manufacturer’s instructions. The concentration of RNA was detected using an RNA/DNA calculator. The RNA was reverse-transcribed to cDNA with the FastKing gDNA Dispelling RT SuperMix (TIANGEN Biotechnology Co., Beijing, China) according to the supplier’s protocol. The relative expression of the target genes was determined by the 2^-ΔΔCT^ method (Zhang et al., 2015). β-actin mRNA was used as an internal control. The relative mRNA concentrations were detected by RT-qPCR using the QuantStudio™ Design & Analysis Software (Thermo Fisher Scientific (China) ltd, Shanghai, China) and the SYBR qPCR SuperMix Plus reagent kit (Novoprotein Technology co., ltd., Suzhou, China). The conditions were as follows: 95°C for 1 min, 95°C, 20 s, and 60°C, 1 min, for 40 cycles. The primer sequences of the genes are shown in **Table 2** (Cui et al., 2020; Lendez et al., 2021).

**Table 2:** Primer sequences of the genes.

Gene	primer sequence (5’-3’)	Length(bp)
TNF-α	F:GGGCTTTACCTCATCTACTCACAG	132
	R:GATGGCAGACAGGATGTTGACC	
IL-1β	F:TGATGACCCTAAACAGATGAAGAGC	134
	R:TGCCCAGGTAGCCATGAATAG	
IL-8	F:ATGACTTCCAAGCTG	238
	R:GGTTTAGGCAGACCTCGTTTC	

### 2.7 Protein extraction and western blotting

Total proteins were extracted using a protein extraction kit (Beyotime Institute of Biotechnology Co., Shanghai, China) according to the manufacturer’s instructions. Protein content was quantified using the BCA protein assay reagent (Beyotime Institute of Biotechnology Co., Shanghai, China), and protein was diluted to the same concentration. Protein (25 μg per lane) was separated using sodium dodecyl sulfate-polyacrylamide gels electrophoresis, and was electro-transferred onto polyvinylidene difluoride (PVDF) membranes. The PVDF membranes were then incubated with either TNF-α and TNFR1 antibodies (Wanleibio Biological Technology Co., Ltd., Shenyang, China), p-IκB α and IκB α antibodies (Cell Signaling Technology, Inc., Danvers, MA, USA), or NF-κB p65 and p-NF-κB p65 antibodies (Abcam, Cambridge, UK). A GAPDH antibody (Affinity Bioscience (China) Co., Hong Kong, China) was used as the internal control. Subsequently the PVDF membranes were incubated with an appropriate secondary antibody (Affinity Bioscience (China) Co., Hong Kong, China). Finally, protein bands were observed using an enhanced chemiluminescent reagent (Biosharp Life Sciences, Hefei, China). Protein grey intensity was quantified by the Fiji/ImageJ-win 64 program normalized GAPDH levels. Each western blot was performed a total of three times.

### 2.8 Statistical analysis

Statistical analyses were performed using GraphPad Prism 8 (GraphPad Software Co., San Diego, CA, USA) for Windows. Analysis of variance was used to evaluate the differences among the groups followed by least significance difference *post-hoc* testing. The results are presented as the mean ± standard deviation (bar on the top of each column). P-values of less than 0.05 were considered significant, and P-values of less than 0.01 were considered markedly significant.

## 3 Results

### 3.1 Intertoe skin explant *F. necrophorum* infection

**Figure 1:**
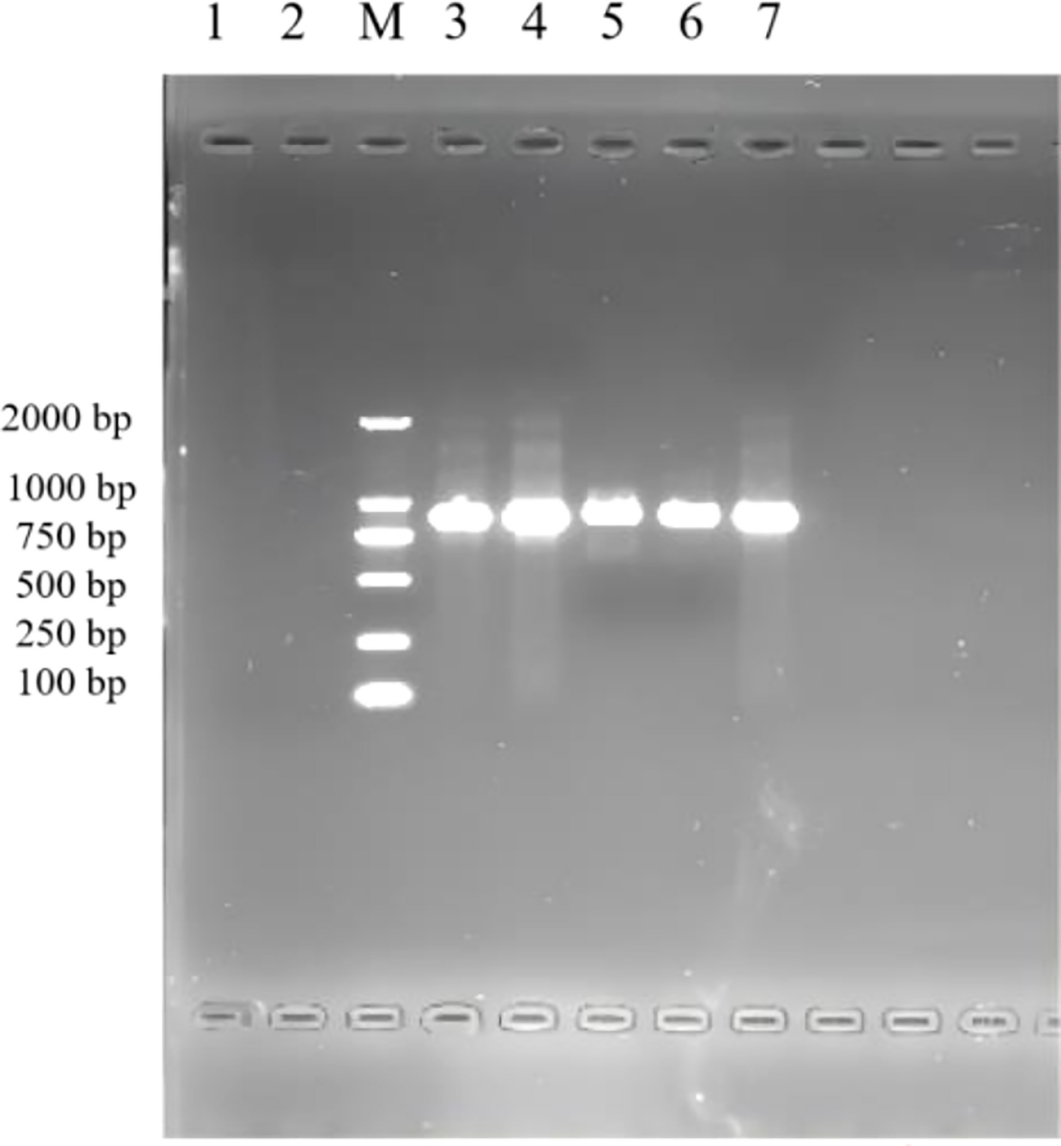
PCR identification of skin explants infected with *F. necrophorum*. 1: negative control; M: DNA Marker DL 2000; 2,3,4,5,6,7: skin explants infected with *F. necrophorum* at 0, 12, 24, 36, 48, and 72 h, respectively.

The results of the 1% agarose gel electrophoresis of PCR products are shown in **Figure 1**. After infection by *F. necrophorum*, skin explants at each culture stage showed amplified bands, indicating that *F. necrophorum* had successfully infected the skin explants.

### 3.2 Pathological changes

**Figure 2:**
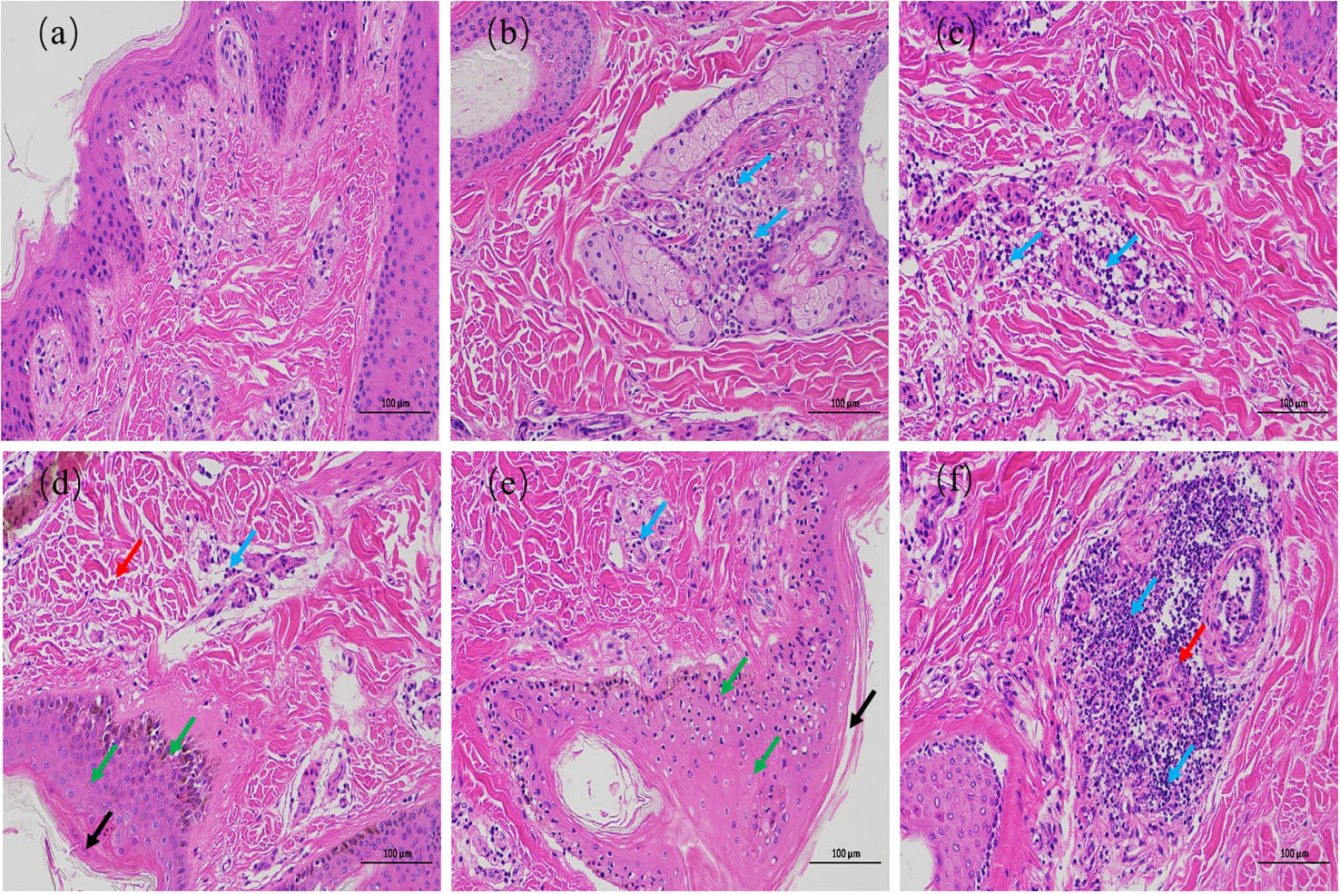
Hematoxylin and eosin staining images of intertoe skin explants infected with *F. necrophorum* over 72 h. **(A)** Skin explants infected with *F. necrophorum* at 0 h, **(B)** 12 h, **(C)** 24 h, **(D)** 36 h, **(E)** 48 h, and **(F)** 72 h. Blue arrows: lymphocyte infiltration in the dermis; Black arrows: hyperkeratosis of the epidermis; Red arrows: hair follicle, sweat gland, and sebaceous gland necrosis; Green arrows: cell degeneration, necrosis, and nuclear pyknosis. (Scale bar = 100 µm) .

The results of HE staining are shown in **Figure 2**. The epidermis of the 0 h control group was intact, and the structure of each layer was clearly visible. The collagen fibers of the dermis were slightly disordered. The muscle fibers of the muscular layer were tightly packed, with scattered hair follicles, sebaceous glands, and sweat glands, and no obvious inflammatory changes were observed (**Figure 2A**). In contrast, the epidermal layer of the skin tissues during the other culture periods showed hyperkeratosis of different degrees, disorganized dermal collagen fibers with different degrees of neutrophil infiltration, and other pathological changes. At 72 h after infection, the skin tissues showed moderate inflammatory reaction, while the other skin tissues showed light inflammatory reaction. At 24 and 36 h after infection, collagen fibers in the dermis of the skin tissue were disordered, and lymphocyte infiltration was observed (**Figures 2C, D**). At 48 and 72 h after infection, the skin tissues showed hyperkeratosis of epithelial cells, thickening of the spinous layer, disordered arrangement of collagen fibers in the dermis, and infiltration of different number of lymphocytes. At 48 h after infection, the skin tissue showed pathological phenomena such as degeneration and necrosis of spinous cells, nuclear pyknosis, and necrosis of a small number of hair follicles and sweat glands (**Figure 2E**). Necrosis of the hair follicle and surrounding sebaceous glands was observed in the skin tissue 72 h after infection, accompanied by a large number of neutrophils, monocytes, and lymphocytes infiltrating the skin, with a strong inflammatory response (**Figure 2F**). These results indicate that the inflammatory response of skin explants increased gradually with increasing duration of *F. necrophorum* infection. The skin explants showed cell degeneration and necrosis 48 h after infection, which would have certain influence on the test results. Therefore, the skin explants at this time was not suitable for *in vitro* study. Consequently, a skin explants model was infected with *F. necrophorum* for 24 hours as the best time point for the follow-up testing.

### 3.3 Results of TUNEL cell apoptosis detection

**Figure 3:**
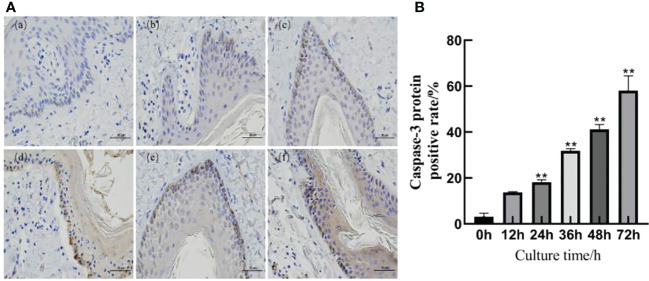
Apoptosis of intertoe explants infected with *F. necrophorum* over 72 h. **(A)** TUNEL staining images of intertoe skin explants infected with *F. necrophorum* over 72 h. (A) Skin explants infected with *F. necrophorum* for 0 h, (B) 12 h, (C) 24 h, (D) 36 h, (E) 48 h, and (F) 72 h. TUNEL stained apoptotic cells are brown, and hematoxylin and eosin stained nuclei are blue. (Scale bar = 50 µm). **(B)** Statistical analysis of the apoptosis rate of skin explants. The data presented are the mean ± SD. *statistical significance (P < 0.05). **statistical significance (P < 0.01). The symbols * and ** indicate the significant differences with the control group.

The TUNEL staining results are shown in **Figure 3A**; a small number of cells were apoptotic in the skin explants of the 0 h control group. At 12, 24, 36, 48, and 72 h after *F. necrophorum* infection, apoptotic cells were significantly expressed in the epidermis and dermis. According to their distribution, the number of apoptotic cells in the epidermis was higher than that in the dermis, and the apoptotic rate was significantly different between groups (**Figure 3B**, P < 0.01). Compared with the control group, the apoptosis rates of skin explants at 12, 24, 36, 48, and 72 h were significantly different (P < 0.01). The TUNEL staining showed that the number of apoptotic cells increased from 3.09% to 58.02% after 72 h *in vitro* culture, and the activity of apoptotic cells increased positively with *in vitro* culture duration.

### 3.4 Expression of Caspase-3 apoptotic protein

**Figure 4:**
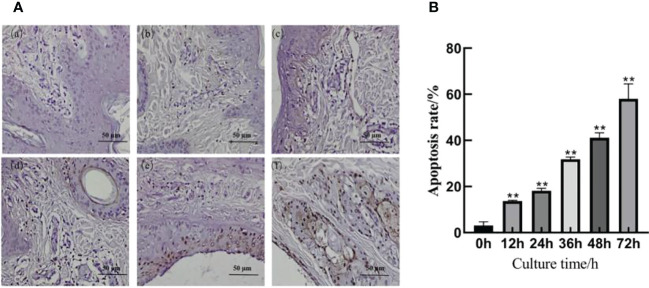
Detection of Caspase-3 protein in skin explants infected with *F. necrophorum* for 72 h. **(A)** Immunohistochemical images of intertoe skin explants infected with *F. necrophorum* over 72 h. (A) Skin explants infected with *F. necrophorum* for 0 h, (B) 12 h, (C) 24 h, (D) 36 h, (E) 48 h, and (F) 72 h. Yellow cells indicate positive expression of Caspase-3 protein by 3, 3’-diaminobenzidine staining. (Scale bar = 50 um). **(B)** Statistical analysis of the apoptosis rate of skin explants. The data presented are the mean ± SD. *statistical significance (P < 0.05). **statistical significance (P < 0.01). The symbols * and ** indicate the significant differences with the control group.

The immunohistochemical results are shown in **Figure 4A**. A slight positive expression of Caspase-3 protein was observed in the skin explants of the 0 h control group. Caspase-3 protein was significantly positively expressed in the epidermis and dermis of the skin explants at 12, 24, 36, 48, and 72 h after *F. necrophorum* infection. According to the distribution, the positive expression of Caspase-3 protein in the epidermis was higher than that in the dermis, and the positive expression rate was significantly different between the groups (P < 0.05). Compared with the control group, there was no significant difference in the positive expression rate of Caspase-3 protein in skin explants at 12 h after infection (**Figure 4B**, P > 0.05). The positive expression rate of Caspase-3 protein in skin explants at 24, 36, 48, and 72 h after infection was significantly different (**Figure 4B**, P < 0.05). Immunohistochemical results showed that the number of apoptotic cells in skin explants increased from 2.49% to 57.61% within 72 h of *in vitro* culture, which was consistent with the TUNEL staining results.

### 3.5 Effect of *F. necrophorum* infection on key proteins of the NF-κB signaling pathway in skin explants

**Figure 5:**
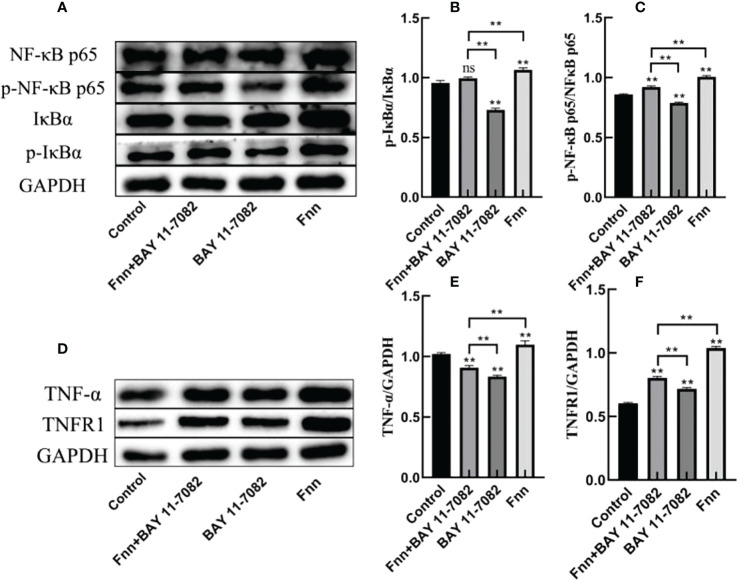
Activation of the nuclear factor-κB (NF-κB) signaling pathway in intertoe explants by *F. necrophorum* infection. Skin explants were treated with *F. necrophorum* a concentration of 1 × 10^8^/mL and/or 50 mM BAY 11-7082 for 24 h. **(A)** Western blotting results of p-NF-κB p65, NF-κB p65, p-IκB α, and IκB α. **(B)** Phosphorylation levels of IκB α. **(C)** Phosphorylation levels of NF-κB p65. **(D)** Western blotting results of TNF-α and TNFR1. **(E)** Significant relationship of TNF-α expression. **(F)** Significant relationship of TNFR1 expression. The data presented are the mean ± SD. *statistical significance (P < 0.05). **statistical significance (P < 0.01). The symbols * and ** indicate the significant differences with the control group. ns, not significant.

The phosphorylation levels of IκBα and NF-κB p65 in the skin explants infected with *F. necrophorum* were significantly higher than those in the control group (**Figures 5A–C**, P < 0.01), while the phosphorylation levels of IκBα and NF-κB p65 in the skin explants treated with an inhibitor were significantly lower than those in the control group (**Figures 5A–C**, P < 0.01). The expression of TNFR1 in skin explants in all groups was significantly higher than that in the control group (**Figures 5D–F**, P < 0.01), while the TNF-α content was affected by the activation of NF-κB signaling pathway, and the TNF-α content in skin explants in the two groups after inhibitor treatment was significantly decreased in the control group (**Figures 5D–F**, P < 0.01). The TNF-α content in skin explants infected with *F. necrophorum* alone was significantly higher than that in the control group (**Figure 5D** - P < 0.01). These results indicate that *F. necrophorum* infection activated the NF-κB signaling pathway in the skin explants.

### 3.6 Effect of *F. necrophorum* infection on mRNA expression and concentration of proinflammatory cytokines in skin explants

**Figure 6:**
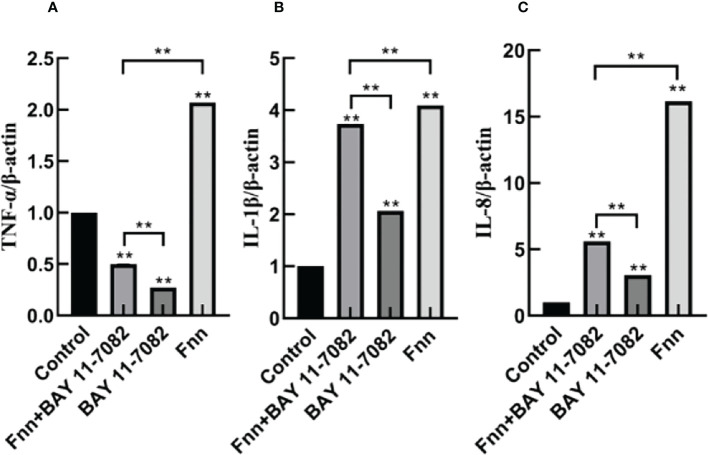
Effect of *F. necrophorum* infection on the mRNA expression of proinflammatory cytokines in intertoe skin explants. **(A–C)** show the mRNA expressions of TNF-α, IL-1β, and IL-8, respectively. The data presented are the mean ± SD. *statistical significance (P < 0.05). **statistical significance (P < 0.01). The symbols * and ** indicate the significant differences with the control group.

**Figure 7:**
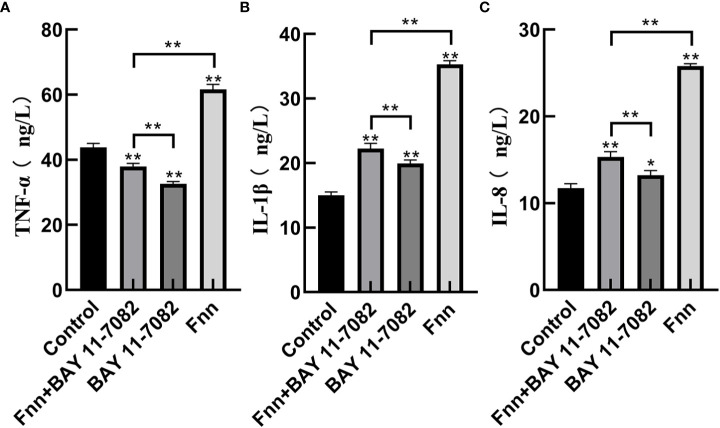
Effect of *F. necrophorum* infection on the concentration of proinflammatory cytokines in intertoe skin explants. **(A–C)** show the concentrations of TNF-α, IL-1β, and IL-8, respectively. The data presented are the mean ± SD. *statistical significance (P < 0.05). **statistical significance (P < 0.01). The symbols * and ** indicate the significant differences with the control group.

The mRNA expression and concentration of TNF-α in the *F. necrophorum* group were significantly higher than in the control group (**Figures 6**, **7A**, P < 0.01), but the expression and concentration of TNF-α were significantly decreased by BAY 11-7082 treatment (**Figures 6**, **7A**, P < 0.01). *F. necrophorum* infection significantly increased the expression of IL-1β and IL-8, even after treatment with BAY 11-7082; the expression and concentration of IL-1β and IL-8 in each group were significantly higher than in the control group (**Figures 6**, **7B, C**, P < 0.01). These results suggest that infection with *F. necrophorum* activated the NF-κB inflammatory pathway, and induced the synthesis and expression of inflammatory cytokines in the intertoe skin explants.

## 4 Discussion

Bovine foot rot is one of the most serious cow limb hoof diseases. It is affected by many pathogenic factors, among which the invasion of pathogenic bacteria is the main cause of this disease. A variety of bacteria including Bacteroides melaninogenicus, Bacteroides fragilis, Staphylococcus aureus, Corynebacterium pyogenes, Proteusbacillus vulgaris, and other conditioned pathogens can cause bovine foot rot (single or mixed infection). With advancing research on bovine foot rot, an increasing number of studies have shown that *F. necrophorum* is the main pathogen and toe space is its main invasion site.

Due to the high experimental costs, research on bovine foot rot has transitioned from the original *in vivo* experiments (Wilson-Welder et al., 2018; Gomez et al., 2012) to a single-layer cell model *in vitro*. However, the latter approach can not reflect the cellular processes *in vivo* due to the lack of multi-cell interactions (Maboni et al., 2017). In addition, there are other drawbacks such as differences in gene and protein expression from normal cells. In this study, a novel research scheme was proposed. An explants model was constructed with cultured cow intertoe skin *in vitro*. Using the complete cow intertoe skin as the research object not only solved the limitations of the single-layer cell model *in vitro*, but also served as the main site of pathogen invasion to more accurately model the occurrence of bovine foot rot. The skin explants model has been developed for decades as an important tool for *in vitro* studies (Randall et al., 2018). During this period, through continuous optimization and improvement, it has become one of the current popular models for replacing animal tests (Bauer et al., 2021), and has played an important role in the study of skin infections caused by bacterial infection (Yang et al., 2013; Lone et al., 2015; Maboni et al., 2017; Ho et al., 2020). It is regarded as the gold standard in the field of *in vitro* skin infection research (Niehues et al., 2018). Expression levels of inflammatory factors were significantly increased in successfully constructed human, mouse, rabbit, and sheep skin infection models, which confirmed the excellent performance of such models in the study of inflammatory response.

The NF-κB signaling pathway plays an important role in mediating the inflammatory response. In the classical pathway, NF-κB is bound to IκBα in the form of a dimer; therefore, the activation conditions depend on the degradation of IκBα. In this study, infection with *F. necrophorum* activated the NF-κB signaling pathway, and phosphorylation of IκBα and proteasome-mediated degradation activated NF-κB and promoted its nuclear transport (Yu et al., 2017), resulting in significantly increased phosphorylation levels of IκBα and NF-κB in the skin explants. The expression of the downstream signaling factor TNF-α and the main receptor TNFR1 also increased significantly. As expected, in the skin explants treated with BAY 11-7082, the inhibition of IκBα phosphorylation resulted in the inhibition of the subsequent NF-κB nuclear transport, and the expression levels of IκBα and NF-κB were significantly decreased, which also resulted in the decrease of the downstream signaling factor TNF-α expression. These results suggest that BAY 11-7082 can reduce the proinflammatory effects of *F. necrophorum* at the signal level. Increased NF-κB activity has been considered as a marker of inflammatory response (Didonato et al., 2012). This study indicates that the inflammatory pathway of NF-κB was activated by the *F. necrophorum* infection of the intertoe skin explants of cows. Therefore, the high expression of the inflammatory pathway of NF-κB may be one of the molecular mechanisms of the inflammatory response of bovine foot rot.

Over activation of the NF-κB inflammatory pathway can significantly increase the expression of inflammatory cytokines (Hu et al., 2016). TNF-α, IL-1β, and IL-18 are proinflammatory cytokines, which are the main markers reflecting inflammation response (Li et al., 2016). Li et al. found that activation of the NF-κB pathway significantly increased the mRNA expression of TNF-α, IL-6, and IL-1β in bovine hepatocytes (Li et al., 2015). In the present study, infection with *F. necrophorum* significantly increased the expression of TNF-α, IL-8, and IL-1β (**Figure 7**), and these inflammatory cytokines further mediated the inflammatory response of the cow intertoe skin. Of note, the expression level of TNF-α, also a downstream inflammatory factor, was lower than that of IL-1β and IL-8. The reason for this is speculated to be that *F. necrophorum* infection not only activated the inflammatory pathway of NF-κB, but also activated the other inflammatory pathways of IL-1β and IL-8 as the main downstream factors. Therefore, the expression level of TNF-α was higher than that of TNF-α.

The specific regulatory mechanism behind the activation of the NF-κB pathway caused by *F. necrophorum* infection needs to be further explored. Runt-related transcription factor 1 (RUNX1) is one of the key regulatory proteins in vertebrates. Studies have found that RUNX1 is involved in embryonic development, tumorigenesis, immune response, hematopoiesis, angiogenesis, and particular inflammatory response (Liu et al., 2015). Recent studies have shown that RUNX1 is highly expressed in lung interstitial and epithelial cells, and plays a key role in lipopolysaccharide-induced pneumonia by regulating the NF-κB pathway (Tang et al., 2018). However, Luo et al. found that in the process of lipopolysaccharide-induced inflammatory response in macrophages, RUNX1 binds to the NF-κB p50 subunit and promotes the expression of inflammatory factors IL-1β and IL-6, while inhibition of transcription activity significantly reduces the level of IL-6 (Luo et al., 2016). Therefore, it is speculated that the activation of the NF-κB inflammatory pathway in cows with hoof rot disease may be closely related to RUNX1. Future research should clarify whether RUNX1 is involved in the inflammation regulation mechanism of anaerobic bacteria infection.

In conclusion, this model of *F. necrophorum* infection in dairy cow intertoe skin explants can be used for the study of bovine foot rot. It revealed that infection of *F. necrophorum* can induce an inflammatory response in the intertoe skin explants, leading to the activation of the inflammatory pathway of NF-κB, and an up-regulation of the expression of inflammatory cytokines TNF-α, IL-8, and IL-1β.

## Data availability statement

The raw data supporting the conclusions of this article will be made available by the authors, without undue reservation.

## Ethics statement

The study protocol was approved by the Ethics Committee on the Use and Care of Animals, Heilongjiang Bayi Agricultural University (Daqing, China). The animal studies were performed in accordance with the Guiding Principles of Animals adopted by the Chinese Association for Laboratory Animal Sciences (DWKJXY2022062).

## Author contributions

JZ designed the project and experiments. HZ, YS, CY, and YY participated in the experiment. HZ analyzed the experimental data and processed the images. All authors contributed to the article and approved the submitted version.

## References

[B1] Allard B. Panariti A. Martin J. G. (2018). Alveolar macrophages in the resolution of inflammation, tissue repair, and tolerance to infection. Front. Immunol. 9, 1777. doi: 10.3389/fimmu.2018.01777 30108592 PMC6079255

[B2] Baker R. G. Hayden M. S. Ghosh S. (2011). NF-κB, inflammation, and metabolic disease. Cell Metab. 13 (1), 11–22. doi: 10.1016/j.cmet.2010.12.008 21195345 PMC3040418

[B3] Bauer T. Gubi D. Klufa J. Novoszel P. Holcmann M. Sibilia M. (2021). Ex-vivo skin explant culture is a model for TSLP-mediated skin barrier immunity. Life (Basel Switzerland) 11 (11), 1237. doi: 10.3390/life11111237 34833113 PMC8623134

[B4] Clifton R. Giebel K. Liu N. L. B. H. Purdy K. J. Green L. E. (2019). Sites of persistence of fusobacterium necrophorum and dichelobacter nodosus: A paradigm shift in understanding the epidemiology of footrot in sheep. Sci. Rep. 9 (1), 14429. 31594981 10.1038/s41598-019-50822-9 PMC6783547

[B5] Cui L. Wang H. Lin J. Wang Y. Dong J. Li J.. (2020). Progesterone inhibits inflammatory response in e.coli- or LPS-stimulated bovine endometrial epithelial cells by NF-κB and MAPK pathways. Dev. Comp. Immunol. 105, 103568. doi: 10.1016/j.dci.2019.103568 31821816

[B6] Didonato J. A. Mercurio F. Karin M. (2012). NF-κB and the link between inflammation and cance. Immunol. Rev. 246 (1), 379–400. doi: 10.1111/j.1600-065X.2012.01099.x 22435567

[B7] Gomez A. Cook N. B. Bernardoni N. D. Rieman J. Dusick A. F. Hartshorn R.. (2012). An experimental infection model to induce digital dermatitis infection in cattle. J. dairy Sci. 95 (4), 1821–1830. doi: 10.3168/jds.2011-4754 22459830

[B8] Ho F. K. Delgado-Charro M. B. Bolhuis A. (2020). Evaluation of an explanted porcine skin model to investigate infection with the dermatophyte trichophyton rubrum. Mycopathologia 185 (2), 233–243. doi: 10.1007/s11046-020-00438-9 32108288

[B9] Hu Z. Song B. Xu L. Zhong Y. Peng F. Ji X.. (2016). Aqueous synthesized quantum dots interfere with the NF-κB pathway and confer anti-tumor, anti-viral and anti-inflammatory effects. Biomaterials 108, 187–196. doi: 10.1016/j.biomaterials.2016.08.047 27639114

[B10] Jiasan Z. Shi S. Cheng X. Chuang X. Hongyou Z. Hongbin W.. (2016). DE-MS based proteomic investigation of dairy cows with footrot. J. Vet. Res. 60 (1), 63–69. doi: 10.1515/jvetres-2016-0010

[B11] Kontturi M. Junni R. Kujala-Wirth M. Malinen E. Seuna E. Pelkonen S.. (2020). Acute phase response and clinical manifestation in outbreaks of interdigital phlegmon in dairy herds. Comp. Immunol. Microbiol. Infect. Dis. 68, 101375. doi: 10.1016/j.cimid.2019.101375 31756638

[B12] Kontturi M. Junni R. Simojoki H. Malinen E. Seuna E. Klitgaard K.. (2019). Bacterial species associated with interdigital phlegmon outbreaks in Finnish dairy herds. BMC Vet. Res. 15 (1), 44. doi: 10.1186/s12917-019-1788-x 30696445 PMC6352363

[B13] Kujala M. Orro T. Soveri T. (2010). Serum acute phase proteins as a marker of inflammation in dairy cattle with hoof diseases. Vet. Rec. 166 (8), 240–241. doi: 10.1136/vr.b4770 20173110

[B14] Lendez P. A. Martinez-Cuesta L. Nieto Farias M. V. Dolcini G. L. Ceriani M. C. (2021). Cytokine TNF-α and its receptors TNFRI and TNFRII play a key role in the *in vitro* proliferative response of BLV infected animals. Vet. Res. Commun. 45 (4), 431–439. doi: 10.1007/s11259-021-09825-z 34453235

[B15] Li X. Huang W. Gu J. Du X. Lei L. Yuan X.. (2015). SREBP-1c overactivates ROS-mediated hepatic NF-κB inflammatory pathway in dairy cows with fatty liver. Cell. signalling 27 (10), 2099–2109. doi: 10.1016/j.cellsig.2015.07.011 26189441

[B16] Li R. Wang J. Wang X. Zhou J. Wang M. Ma H.. (2016). Increased βTrCP are associated with imiquimod-induced psoriasis-like skin inflammation in mice *via* NF-κB signaling pathway. Gene 592 (1), 164–171. doi: 10.1016/j.gene.2016.07.066 27476970

[B17] Liu H. P. Cao A. T. Feng T. Li Q. Zhang W. Yao S.. (2015). TGF-β converts Th1 cells into Th17 cells through stimulation of Runx1 expression. Eur. J. Immunol. 45 (4), 1010–1018. doi: 10.1002/eji.201444726 25605286 PMC4441226

[B18] Lone A. G. Atci E. Renslow R. Beyenal H. Noh S. Fransson B.. (2015). Staphylococcus aureus induces hypoxia and cellular damage in porcine dermal explants. Infect. Immun. 83 (6), 2531–2541. doi: 10.1128/IAI.03075-14 25847960 PMC4432762

[B19] Luo M. C. Zhou S. Y. Feng D. Y. Xiao J. Li W. Y. Xu C. D.. (2016). Runt-related transcription factor 1 (RUNX1) binds to p50 in macrophages and enhances TLR4-triggered inflammation and septic shock. J. Biol. Chem. 291 (42), 22011–22020. doi: 10.1074/jbc.M116.715953 27573239 PMC5063984

[B20] Maboni G. Davenport R. Sessford K. Baiker K. Jensen T. K. Blanchard A. M.. (2017). A novel 3D skin explant model to study anaerobic bacterial infection. Front. Cell. Infect. Microbiol. 7. doi: 10.3389/fcimb.2017.00404 PMC5604072 28959685

[B21] Mitchell J. P. Carmody R. J. (2018). NF-κB and the transcriptional control of inflammation. Int. Rev. Cell Mol. Biol. 335, 41–84. doi: 10.1016/bs.ircmb.2017.07.007 29305014

[B22] Niehues H. Bouwstra J. A. El Ghalbzouri A. Brandner J. M. Zeeuwen P. L. J. M. van den Bogaard E. H. (2018). 3D skin models for 3R research: The potential of 3D reconstructed skin models to study skin barrier function. Exp. Dermatol. 27 (5), 501–511. doi: 10.1111/exd.13531 29518287

[B23] O'Driscoll K. McCabe M. Earley B. (2015). Differences in leukocyte profile, gene expression, and metabolite status of dairy cows with or without sole ulcers. J. Dairy Sci. 98 (3), 1685–1695. doi: 10.3168/jds.2014-8199 25557893

[B24] Randall M. J. Jüngel A. Rimann M. Wuertz-Kozak K. (2018). Advances in the biofabrication of 3D skin *in vitro*: Healthy and pathological models. Front. Bioeng. Biotechnol. 6. doi: 10.3389/fbioe.2018.00154 PMC6220074 30430109

[B25] Sy J. Ang L. C. (2019). Microtomy: Cutting formalin-fixed, paraffin-embedded sections. Methods Mol. Biol. (Clifton NJ) 1897, 269–278. doi: 10.1007/978-1-4939-8935-5_23 30539451

[B26] Tang X. Sun L. Wang G. Chen B. Luo F. (2018). RUNX1: A regulator of NF-kB signaling in pulmonary diseases. Curr. Protein Pept. Sci. 19 (2), 172–178. 28990531 10.2174/1389203718666171009111835 PMC5876917

[B27] Van Metre D. C. (2017). Pathogenesis and treatment of bovine foot rot. Vet. Clin. North Am. Food Anim. Pract. 33 (2), 183–194. doi: 10.1016/j.cvfa.2017.02.003 28579042

[B28] Wilson-Welder J. H. Nally J. E. Alt D. P. Palmer M. V. Coatney J. Plummer P. (2018). Experimental transmission of bovine digital dermatitis to sheep: Development of an infection model. Vet. Pathol. 55 (2), 245–257. doi: 10.1177/0300985817736572 29145798

[B29] Yang Q. Phillips P. L. Sampson E. M. Progulske-Fox A. Jin S. Antonelli P.. (2013). Development of a novel *ex vivo* porcine skin explant model for the assessment of mature bacterial biofilms. Wound Repair regeneration Off. Publ. Wound Healing Soc. [and] Eur. Tissue Repair Soc. 21 (5), 704–714. doi: 10.1111/wrr.12074 23927831

[B30] Yu L. Li L. Medeiros L. J. Young K. H. (2017). NF-κB signaling pathway and its potential as a target for therapy in lymphoid neoplasms [J]. Blood Rev. 31 (2), 77–92. doi: 10.1016/j.blre.2016.10.001 PMC5382109 27773462

[B31] Zhang M. Zhang S. Hui Q. Lei L. Du X. Gao W.. (2015). β-hydroxybutyrate facilitates fatty acids synthesis mediated by sterol regulatory element-binding Protein1 in bovine mammary epithelial cells. Cell. Physiol. Biochem. Int. J. Exp. Cell. Physiol. Biochem. Pharmacol. 37 (6), 2115–2124. doi: 10.1159/000438569 26599760

